# Synthesis of magnetic nanocarbon using palm oil as the green precursor via microwave-assisted arc for wastewater treatment

**DOI:** 10.1038/s41598-022-21982-y

**Published:** 2022-11-04

**Authors:** Nurul Zariah Jakaria Zakaria, Shaifulazuar Rozali, Nabisab Mujawar Mubarak, Mohammad Khalid

**Affiliations:** 1 Department of Mechanical Engineering, Faculty of Engineering, University of Malaya, 50603 Kuala Lumpur, Malaysia; 2 Petroleum and Chemical Engineering, Faculty of Engineering, Universiti Teknologi Brunei, Bandar Seri Begawan, BE1410 Brunei Darussalam; 3 Graphene and Advanced 2D Materials Research Group (GAMRG), School of Engineering and Technology, Sunway University, No. 5, Jalan University, Bandar Sunway, 47500 Subang Jaya, Selangor Malaysia

**Keywords:** Environmental chemistry, Environmental sciences, Environmental social sciences, Materials science

## Abstract

The presence of metal with microwave irradiation has always invited controversial arguments as the metal will catch on fire easily. But interestingly, researchers found that arc discharge phenomena provide a promising way for molecule cracking to synthesize nanomaterials. This study developed a single-step yet affordable synthesis approach that combines microwave heating and arcing in transforming crude palm oil into magnetic nanocarbon (MNC), which can be considered a new alternative for the palm oil sectors. It involves synthesizing the medium at a partial inert condition with constant coiled stainless steel metal wire (dielectric media) and ferrocene (catalyst). This approach successfully demonstrates heating at a temperature ranging from 190.9 to 472.0 °C with different synthesis times (10–20 min). The produced MNC shows formations of spheres with average sizes of 20.38–31.04 nm, mesoporous structure (SBET: 14.83–151.95 m^2^/g), and high content of fixed carbon (52.79–71.24wt%), and the ratio of the D and G bands (*I*_D_/*I*_G_) is 0.98–0.99. The formation of new peaks in the FTIR spectra (522.29–588.48 cm^−1^) supports the appearance of the FeO compounds from the ferrocene. The magnetometer shows high magnetization saturation (22.32–26.84 emu/g) in ferromagnetic materials. The application of the MNC in wastewater treatment has been demonstrated by evaluating their adsorbent capability with Methylene Blue (MB) adsorption test at a different concentrations varying between 5 and 20 ppm. The MNC produced at synthesis time (20 min) shows the highest adsorption efficiency (10.36 mg/g) compared to others, with 87.79% removal of MB dye. As a result, the value for Langmuir is not promising compared to Freundlich, with *R*^2^ being around 0.80, 0.98, and 0.99 for MNC synthesized at 10 min (MNC10), 15 min (MNC15), and 20 min (MNC20), respectively. Hence, the adsorption system is in a heterogeneous condition. The microwave-assisted arcing thereby presents a promising approach to transforming CPO into MNC that could remove the hazardous dye.

## Introduction

Microwave irradiation can heat the most inner of the materials via molecular interaction of the electromagnetic field^1^. This microwave reaction is unique as it promotes fast and homogenous thermal reactions. Consequently, the heating process can be accelerated and enhance the chemical reactions^2^. At the same time, the microwave reaction can end up with high purity and yield products due to shorter time reactions^3,4^. Due to its fascinating properties, microwave irradiation promotes interesting microwave-assisted synthesis used in many studies, including chemical reactions and nanomaterials synthesis^5,6^. During the heating process, the dielectric properties of the receptor inside the medium play an essential role as it will raise a hot spot in the medium that could produce different morphology and properties of nanocarbon^7^. A study by Omoriyekomwan et al. produced hollow carbon nanofibres from palm kernel with activated carbon and nitrogen flow^8^. Besides that, Foo and Hameed determined the use of catalysts in creating activated carbon from oil palm fibers inside a microwave at 350 W^9^. Therefore, it is possible to offer a similar method for converting crude palm oil to produce MNC by introducing suitable receptors.

An interesting phenomenon has been observed between microwave irradiations and metals with sharp edges, tips, or submicroscopic irregularities^10^. The presence of both entities will be subjected to an electric arc or spark (generally referred to as arc discharge)^11,12^. The arcing will support the formation of more local hotspots and affect the reaction, thus enhancing the medium's chemical composition^13^. This special yet interesting phenomenon has attracted various studies such as in pollutant removal^14,15^, biomass tar cracking^16^, microwave-assisted pyrolysis^17,18^ as well as materials synthesis^19–21^.

Recently, nanocarbons such as CNTs, carbon nanosphere, and decorated reduced graphene oxide^22^ are gaining attention due to their properties. These nanocarbons have great potential in various applications, from power generation to purification or remediation of water pollution^23^. Moreover, there are demands for excellent carbonaceous properties but at the same time required good magnetism properties. This is useful for multi-function applications, including high adsorption of metal ions and dyes in wastewater treatment, magnetic modifiers in biofuel, or even efficient microwave absorbers^24–28^. At the same time, these carbons have another advantage, including an increase in the samples' surface area of active sites.

In recent years, the studies for magnetic nanocarbon materials have been growing vividly. Generally, these magnetic nanocarbons are multi-function materials containing nano-sized magnetic materials to trigger the response from external catalysts such as external static or an alternating magnetic field^29^. Due to their magnetic properties, the magnetic nanocarbons can combine with a vast range of active components and complex structures for immobilization^30^. At the same time, magnetic nanocarbon (MNC) exhibits excellent efficiency in the adsorption of contaminants from aqueous solutions. Furthermore, high specific surface areas and pores developed in the MNC could increase adsorption capacity^31^. A magnetic separator can separate the MNC from a high reaction solution, making it a feasible and manageable adsorbent^32^.

Several investigators have demonstrated that high-quality nanocarbon can be obtained using crude palm oil palm^33,34^. Palm oil, scientifically known as *Elais Guneensis,* is recognized as one of the essential edible oils contributing around 76.55 million metric tonnes of production in 2021^35^. The crude palm oil or CPO contained a balanced ratio of unsaturated fatty acids (UFA) and saturated fatty acids (SFA). Most of the hydrocarbon in the CPO is a triglyceride, a glycerol ester consisting of three triglyceride acetous contents and one glycerol content^36^. These hydrocarbons could be summed up into huge carbon content, becoming a potential green precursor in producing nanocarbon^37^. Based on the literature, CNTs^37–40^, carbon nanospheres^33,41^, and graphene^34,42,43^ are commonly synthesized using crude palm oil or cooking grade oil. These nanocarbons have great potential in various applications, from power generation to purification or remediation of water pollution.

Thermal-based synthesis such as CVD^38^ or pyrolysis^33^ becomes the favourable method for palm oil decomposition. Unfortunately, high temperatures during the process will increase the production cost. Lengthy, tedious procedures and purification methods are needed to obtain the preferable materials^44^. However, the need for physical separation and cracking is undeniable as crude palm oil has good stability at high temperatures^45^. Thus, a higher temperature is still needed to convert crude palm oil into carbonaceous materials. Liquid arcing could be considered the best potential and novel method for synthesizing magnetic nanocarbon^46^. The methods provide direct energy to the precursor and solutions at a highly excited energy state. The arcing could initiate the cracking of the carbon bonds in crude palm oil. However, the electrode spacings used may have to abide by strict requirements that will bring limitations on an industrial scale and thus, developing an effective method remains.

To our knowledge, research in microwave-assisted arcing as a method of synthesizing nanocarbon is limited. At the same time, the usage of crude palm oil as the precursor is not fully explored yet. Hence, this study aim and investigates the potential of producing magnetic nanocarbons from crude palm oil precursors via microwave-assisted arcing. The abundance of palm oil should be manifested into new products and applications. This new method of transforming palm oil could help raise the economic sectors and be another source of income for the palm oil industries, especially for the affected small holder’s palm oil plantation. According to a study on smallholders in Africa done by Ayompe et al., smallholders only make more money when they process their fresh fruit bunches by themselves and sell the CPO rather than selling them to intermediaries, which are costly and a tedious work^47^. At the same time, the number of factories that are closing increases day by day, affecting the products from palm oil-based applications due to COVID-19. Interestingly, as the microwave is available in most households, and the proposed method in this research can be considered feasible and affordable, the production of the MNC can be considered another alternative for the small holder’s palm oil plantation. Meanwhile, on a bigger scale, companies can invest in a big-scale reactor that produces large-scale MNC.

This study mainly covers the synthesis process using stainless steel as dielectric media with different duration times. Most general studies involving microwave and nanocarbon present an acceptable synthesis time of 30 min or more^33,34^. In supporting an affordable and feasible idea that is practical, this study aims to achieve the production of the MNC below the average of synthesis time. At the same time, the research portrays the technology readiness level of 3 as the theory is proved and conducted on a lab scale. Later, the obtained MNCs were characterized by their physical, chemical and magnetic properties. Then, Methylene Blue is used to demonstrate the adsorption capability of the produced MNCs.

## Materials and method

### Materials

Crude palm oil was obtained from Apas Balung Mill, Sawit Kinabalu Sdn. Bhd., Tawau and used as the carbon precursor for the synthesis. Meanwhile, the stainless-steel metal wire with a diameter of 0.90 mm was used as the dielectric media. Ferrocene (99% purity) obtained from Sigma-Aldrich, USA, was chosen as the catalyst in this study. Methylene Blue (Bendosen, 100 g) is later used in the adsorption experiments.

### Microwave reactor set up

This study has modified a home microwave oven (Panasonic: SAM-MG23K3513GK) as a microwave reactor. Three holes were fabricated at the top of the microwave oven for the gas inlet, outlet, and thermocouple. The thermocouple probe was insulated with ceramic tubing and placed in the same condition every time the experiment prevented an unwanted accident. Meanwhile, a borosilicate glass reactor with a three-hole lid was used to hold the samples and gas tubes. The schematic diagram of the microwave reactor can be referred to in Supplementary Fig. 1.

### Microwave-assisted arc in the liquid synthesis

Synthesizing magnetic nanocarbon was carried out using crude palm oil as the carbon precursor and ferrocene as the catalyst. An approximate 5 wt.% of ferrocene catalyst was prepared by floated catalyst method. The ferrocene was mixed with 20 ml of crude palm oil for 60 rpm at 30 min. Then, the mixture was transferred into an alumina crucible, and a 30 cm stainless wire was coiled and placed vertically inside the crucible. The alumina crucible is fed into the glass reactor and firmly established inside the microwave with a sealed glass lid. Before reactions, nitrogen gas is purged into the chamber for 5 min to remove unwanted air inside the chamber. The power of the microwave is increased to 800 W as it is the maximum power supply of the microwave, which could support a good arcing. Hence, it could promote a good condition of a synthesis reaction. At the same time, it is the common range of watt power used in microwave synthesis reactions^48,49^. The mixture is heated for 10, 15 or 20 min during the reaction. Once the reaction is completed, the reactor and microwave are allowed to cool at room temperature naturally. The final product inside the alumina crucible is black precipitates with the coiled wire.

The black precipitates were collected and washed alternately with ethanol, isopropyl alcohol (70%), and distilled water several times. After washing and purifying, the products are placed inside a conventional oven to dry overnight at 80 °C to evaporate unwanted impurities. The products are collected afterward for characterization. The samples are labeled MNC10, MNC15, and MNC20 for the magnetic nanocarbon synthesized for 10 min, 15 min, and 20 min.

### Characterisation of magnetic nanocarbon

#### Morphological characterizations

The morphologies of the MNC are observed using field-emission scanning electron microscopy, or FESEM (Model Zeiss Auriga), with magnification ranging from 100 to 150kX. Meanwhile, Energy Dispersive X-ray Spectroscopy (EDS) analyzed elemental composition. The EDS analysis was done at a 2.8 mm working distance with 1 kV accelerating voltage. The specific surface areas and pore values of the MNC are measured using Brunauer–Emmett–Teller (BET) method, including the adsorption–desorption isotherm N_2_ at 77 K. The analysis is performed with a model surface area meter (MICROMERITIC ASAP 2020).

#### Chemical bonds analysis

The crystallinity and phases of the magnetic nanocarbons are identified using powder X-ray Diffraction or XRD (Burker D8 Advance) with *λ* = 0.154 nm. The diffraction patterns were recorded between 2*θ* = 5 and 85° with a scanning rate of 2° min^−1^. In addition, the chemical structure of MNC is examined by Fourier Transform Infrared Spectroscopy (FTIR). Analysis was done using Perkin Elmer FTIR-Spectrum 400 with a scanned rate ranging from 4000 to 400 cm^−1^. In examining the structural characteristic of the magnetic nanocarbons, Raman spectroscopy was performed using a Neodymium doped laser (532 nm) in U-RAMAN spectroscopy with a 100X objective lens.

#### Magnetic properties analysis

A vibrating sample magnetometer or VSM (Lake Shore 7400 series) is used to measure the magnetic saturation of iron oxide in the MNCs. The magnetic field used is around 8 K Oe with 200 points acquisition.

### Adsorption experiments

In studying the potential of MNC as an adsorbent, the cation dye Methylene Blue (MB) was employed for the adsorption experiments. The MNC (20 mg) was added to 20 ml of the aqueous solution of Methylene Blue in the standard concentration range of 5–20 mg/L^50^. The solution's pH was set to a neutral pH of 7 throughout the study. The solutions were mechanically agitated in a rotary shaker (Lab Companion: SI-300R) at 150 rpm at 303.15 K. The MNCs were then separated using a magnet. The concentration of MB solution before and after the adsorption experiment was observed using a UV–Vis spectrophotometer (Varian Cary 50 UV–Vis Spectrophotometer), referring to the standard curve of Methylene Blue at a maximum wavelength of 664 nm. The experiments were repeated three times, and the means values were presented. The removal amount of the MB from the solutions was calculated using the general equations of the amount of MB adsorbed at equilibrium, *qe*, and the percentage removal, %.

The adsorption isotherm experiments were carried out as well by agitating MB solution at different concentrations of (5–20 mg/l) with 20 mg of adsorbent at a constant temperature of 293.15 K. The experiments were conducted in a 150 ml conical flask with 20 ml of dye solution and 20 mg of adsorbent dosage for all MNCs.

## Results and discussion

### Morphological and structural features of magnetic nanocarbon spheres

Numerous studies on iron and magnetic carbons have been done in the past few decades. These carbon-based materials with magnetic properties have increasing attention due to their magnificent electrical and magnetic properties, which led to various potential technological applications, mainly in electrical appliances and water treatment. This study synthesized nanocarbons by cracking hydrocarbons in crude palm oil by microwave discharge. The synthesis was done at different times, between 10 to 20 min at a fixed ratio of precursor and catalyst (5:1), using metal susceptor (coiled SS) and partially inert (with the flow of nitrogen at the beginning of the experiment to purge out unwanted air). The produced carbonaceous deposits were in the form of black solid powdery, as shown in Supplementary Fig. 2a. The deposited carbon yields were around 5.57, 8.21, and 11.67% for the synthesis times of 10 min, 15 min, and 20 min, respectively. This condition indicates that longer synthesis time contributed to higher yields^51^—low yield, most probably due to the short reaction time and catalyst activity.

Meanwhile, the synthesis temperature vs. time graph for the produced nanocarbons can be referred to in Supplementary Fig. 2b. The highest temperature obtained was 190.9 °C, 434.5 °C, and 472 °C each for MNC10, MNC15, and MNC20. A steep slope can be seen for each curve, indicating a constant temperature increment inside the reactor due to the heat generated during metal arcing. This can be seen at 0–2 min, 0–5 min, and 0–8 min for MNC10, MNC15, and MNC20, respectively. After reaching specific points, the slope continued to hover until the highest temperature with a moderate gradient.

#### Field emission scanning electron microscopy analysis

**Figure 1:**
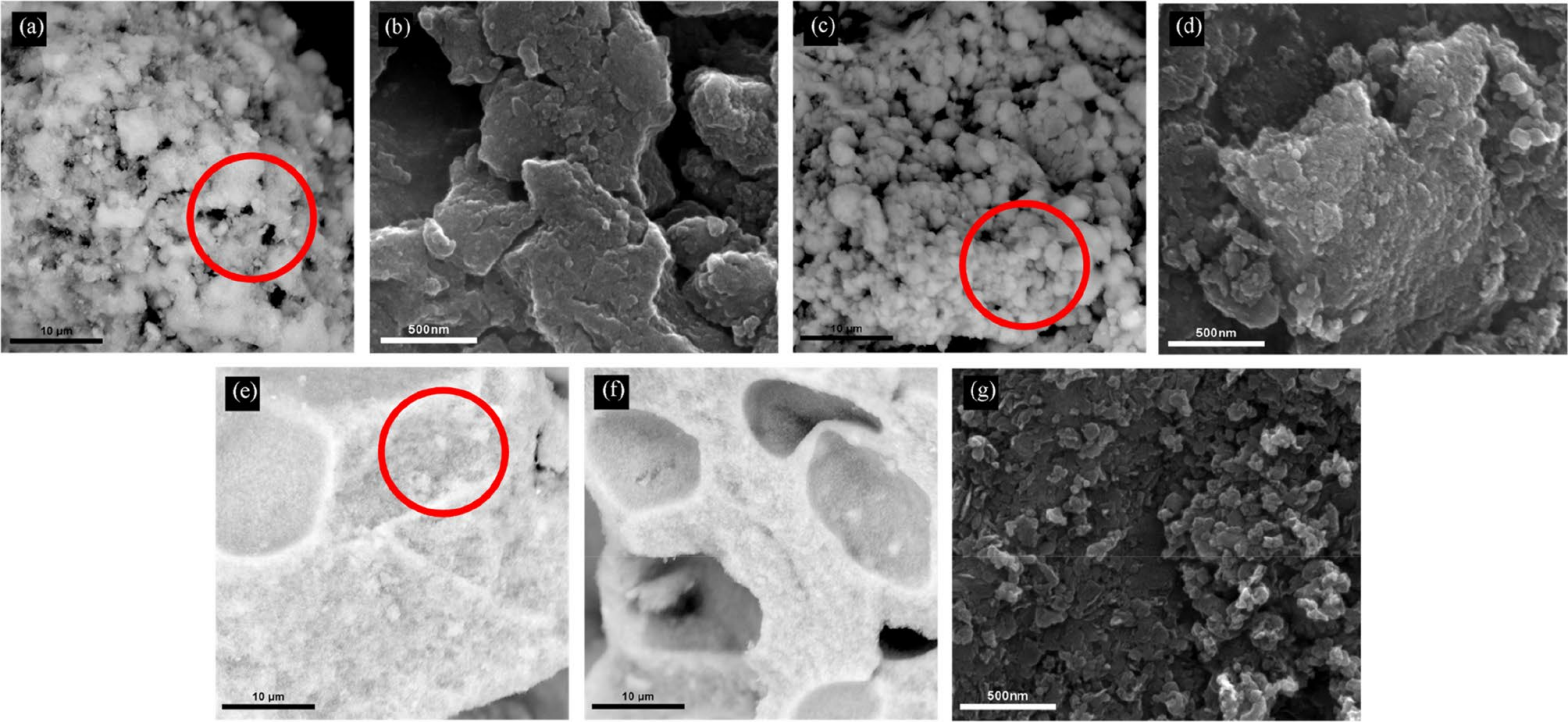
FESEM images of the nanocarbon synthesized for (**a**, **b**) 10 min, (**c**, **d**) 15 min, and (**e**–**g**) 20 min at 7,000X and 15,000X magnification.

Field emission scanning electron microscopy (FESEM) was used to observe the surface morphology of the MNC samples. As shown in Fig. 1, magnetic nanocarbon exhibited a slightly different morphological structure at other synthesis times. The FESEM images of MNC10 in Fig. 1a,b show the formation of carbon spheres comprised of entangled and attached micro and nano-sized spheres due to the high surface tension. At the same time, the presence of Van der Waals forces leads to the agglomerated collection of carbon spheres^52^. Allowing more time during the synthesis resulted in a smaller size and increased number of spheres due to a more extended cracking reaction. Figure 1c shows MNC15 has almost a perfect sphere shape. However, agglomerated spheres could still be seen forming mesopores, which later can be a good spot for adsorbing Methylene Blue. Under a high magnification of 15,000X in Fig. 1d, more agglomeration of carbon spheres can be seen with average sizes of 20.38 nm.

In Fig. 1e–g, the MNC20 portrays the development of pores with small spheres on the magnetic carbons' surface and reassembles the magnetic activated carbon morphology^53^. The pores are developed randomly on the surface of the magnetic carbons with various diameters and widths. Thus, this might explain why MNC20 displayed a larger surface area and pore volume, as shown in the BET analysis, as more pores were created on its surface than at other synthesis times. The micrographs taken under a high magnification of 15,000X show that the sizes are not homogenous with irregular particle shapes, as shown in Fig. 1g. More agglomerated spheres are formed by increasing the growth time to 20 min.

Interestingly, a few distorted carbon flakes also could be found at the same spot. The diameters of the spheres range from 5.18 to 96.36 nm. This formation may be due to high temperature and microwave that promotes the appearance of different nucleation^54^. The estimated sphere sizes of the prepared MNCs are on average 20.38 nm for MNC10, 24.80 nm for MNC15, and 31.04 nm for MNC20. The size distributions of the spheres are shown in Supplementary Fig. 3.

#### Energy-dispersive X-ray analysis (EDS Analysis)

Supplementary Fig. 4 shows the EDS spectrum and the summary of the elemental composition for MNC10, MNC15, and MNC20, respectively. Based on the spectrum, it was noticed that each nanocarbon contained different amounts of C, O, and Fe. This happened due to varying oxidation and cracking reactions during additional synthesis times. It was believed that a high amount of C originated from the carbon precursor, which is crude palm oil. Meanwhile, the low percentage of O is due to the oxidation process during the synthesis period. At the same time, Fe has been attributed to the iron oxide precipitated on the nanocarbon surface originating from the breakdown of ferrocene. Other than that, Supplementary Fig. 5a–c show the elemental mapping of MNC10, MNC15, and MNC20. Based on the basic mapping, it was observed that the Fe is well dispersed on top of the MNC surface.

#### Brunauer–Emmett–Teller (BET) analysis

**Figure 2:**
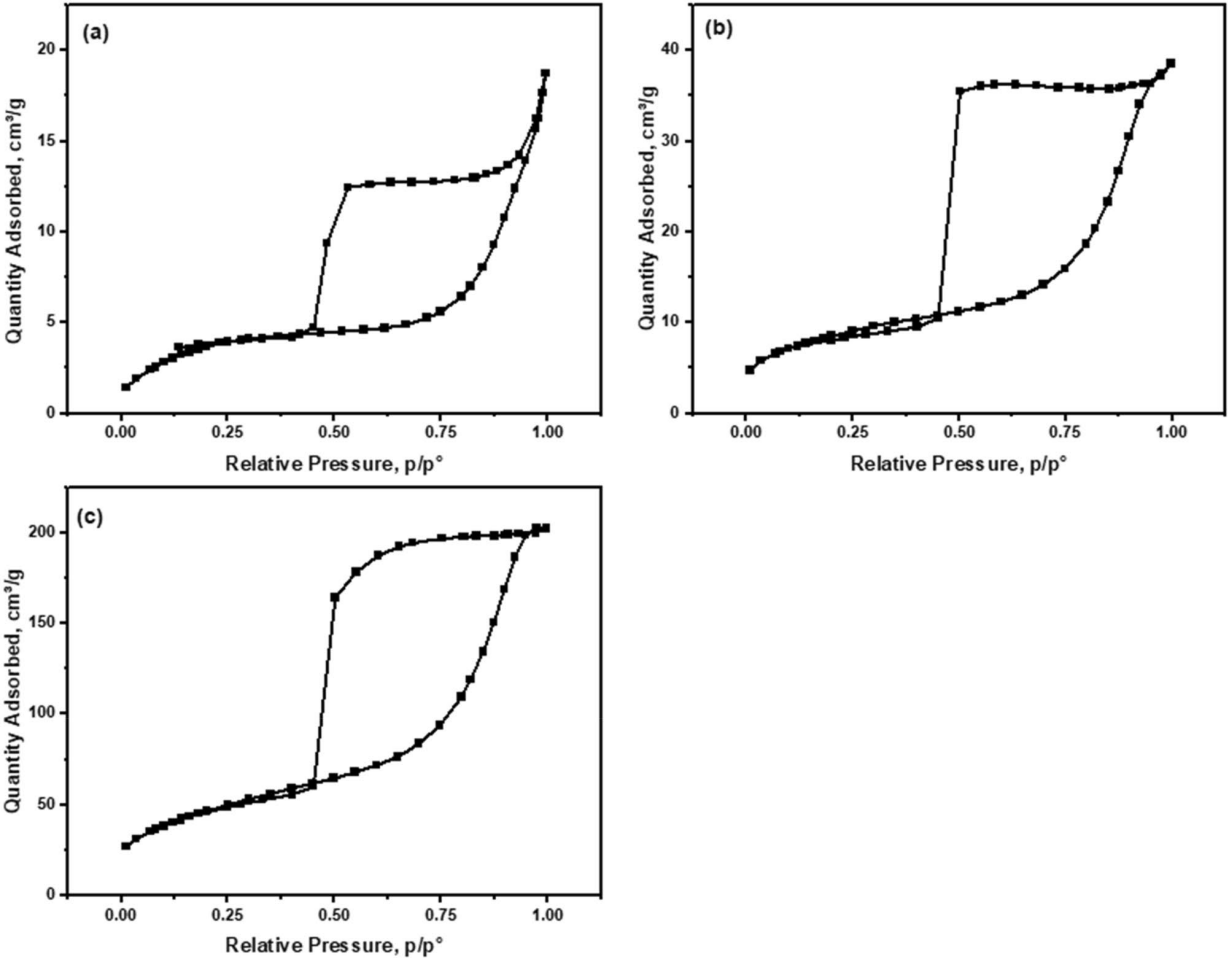
Magnetic hysteresis of (**a**) CMN10, (**b**) CMN15, and (**c**) CMN20.

The nitrogen adsorption–desorption analysis provides information on the materials' adsorption mechanisms and porous structure. The N_2_ adsorption isotherm and the BET surface plot for the MNC are shown in Fig. 2. Based on the FESEM images, and it was expected that the adsorption behavior would exhibit a combination of a microporous-mesoporous structure due to the aggregation. However, the graphs in Fig. 2 show that the adsorbents resemble Type IV isotherms and hysteresis loop of type H2 according to the IUPAC^55^. This type of isotherm often resembles mesoporous materials. The adsorption behaviour in mesoporous is usually determined by the adsorbent-adsorptive reaction and the interactions between the molecules in the condensed state. The sigmoidal or S-shaped adsorption isotherm is typically caused by monolayer-multilayer adsorption followed by a phenomenon whereby the gas will condense to a liquid phase in a pore at a pressure less than the saturation pressure of the bulk liquid, which is known as pore condensation^56^. The capillary condensation in the pores occurs at a relative pressure (*p/p*_*o*_) above 0.50. Meanwhile, the type H2 hysteresis is exhibited by complex pore structures attributed to pore-bocking or percolation in a narrow range of pore necks.

The surface physical parameters obtained from the BET testing are summarized in Table 1. The BET surface area and total pore volume were significantly improved with increasing synthesis time. The average pore diameter was 7.2779 nm, 7.6275 nm, and 7.8223 nm for MNC10, MNC15, and MNC20. Based on the IUPAC recommendation, these moderate pores can be classified as mesoporous material. The mesoporous structure could make the Methylene Blue easier to penetrate and adsorbed by MNC^57^. The highest synthesis time (MNC20) shows the highest surface area, followed by MNC15 and MNC10. Higher BET surface area could enhance the adsorption performance as more surface-active sites are available.

**Table 1:** SBET parameters for different MNC.

MNC	Total surface area (BET) m^2^g^−1^	Total pore volume cm^3^g^−1^	Average pore diameter, Å
MNC10	14.8257	0.028993	78.2232
MNC15	31.2851	0.059657	76.2748
MNC20	161.9492	0.312881	72.7786

### Chemical analysis of magnetic nanocarbons

#### X-ray diffraction (XRD) analysis

**Figure 3:**
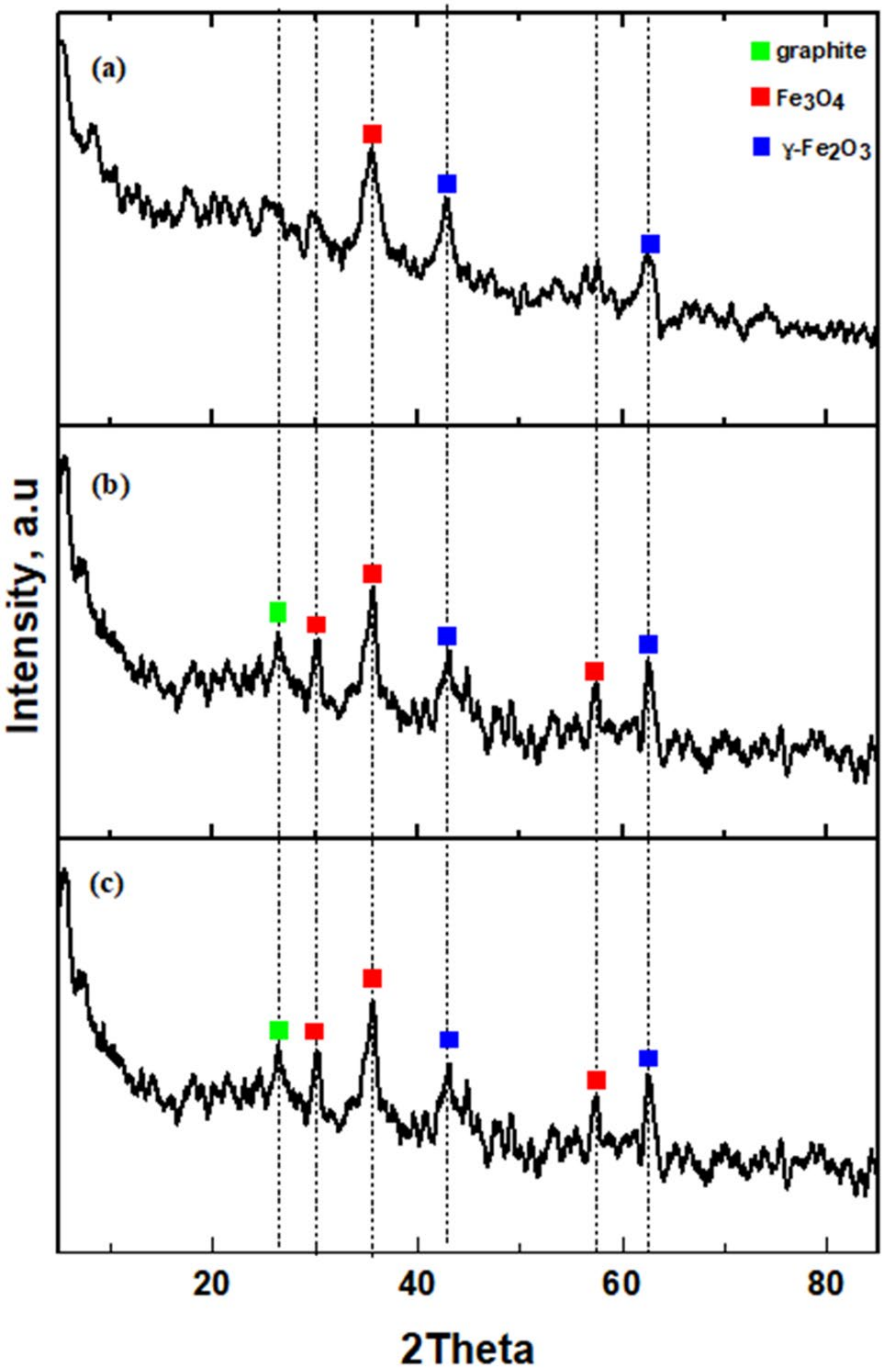
X-ray diffraction patterns of (**a**) MNC10, (**b**) MNC15, and (**c**) MNC20.

XRD patterns of the synthesized MNCs are shown in Fig. 3. At high temperature, the ferrocene experiences cracking too and form iron oxide. Figure 3a illustrates the XRD diffractogram pattern of MNC10. It shows two peaks of 2*θ*: 43.0° and 62.32°, which are assigned for ɣ-Fe_2_O_3_ (JCPDS #39–1346). Meanwhile, a tense peak at 2*θ*: 35.27° is given for Fe_3_O_4._ On the other hand, the diffractogram of the MNC15 in Fig. 3b shows new peaks, most probably due to the increase in temperature and synthesis time. Although the peak 2*θ*: 26.202° was less tense, the diffractogram pattern matches with the JCPDS file of graphite (JCPDS #75–1621), which shows the presence of graphitic crystalline inside the nanocarbons. This peak is absent in MNC10, probably due to low arcing temperature during the synthesis time. The presence of 3 tense peaks at 2*θ*: 30.082°, 35.502°, 57.422° are assigned to Fe_3_O_4_. It also displays two peaks indicating the presence of ɣ-Fe_2_O_3_ at 2θ: 43.102° and 62.632°. For the MNC synthesized at 20 min (MNC20), shown in Fig. 3c, a similar diffractogram pattern could be observed in MNC15. The peak of graphite at 26.382° also could be observed in the MNC20. Three sharp peaks illustrated at 2*θ*: 30.102°, 35.612°, 57.402° are assigned to Fe_3_O_4_. Additionally, the presence of ɣ-Fe_2_O_3_ is depicted at 2*θ*: 42.972° and 62.61. The presence of iron oxide compounds in the produced MNC may positively impact the ability to adsorb Methylene Blue later.

#### FTIR analysis

Characteristics of the chemical bonds in MNC and crude palm oil samples were identified from the FTIR reflectance spectrum in Supplementary Fig. 6. Initially, six significant peaks in crude palm oil represent four different chemical compositions, referred to in Supplementary Table 1. The essential peaks identified inside the CPO are 2913.81 cm^−1^, 2840 cm^−1^, and 1463.34 cm^−1^, which are assigned to the CH stretching vibration of alkane and other CH_2_ or CH_3_ aliphatic groups. The identified peaks foresters are 1740.85 cm^−1^ and 1160.83 cm^−1^. Peak 1740.85 cm^−1^presents the C=O bond for esters carbonyl stretching of the functional groups of triglycerides. Meanwhile, the peak of 1160.83 cm^−1^ is the fingerprint for stretching the C–O ester group^58,59^. At the same time, the peak of 813.54 cm^−1^ represents the alkane group's fingerprints.

Consequently, some absorbance peaks in crude palm oil disappear as the synthesis time increases. The peaks 2913.81 cm^−1^ and 2840 cm^−1^ can still be observed in MNC10, but interestingly, in MNC15 and MNC20, the peaks tend to disappear due to oxidation. At the same time, the FTIR analyses of the magnetic nanocarbon show new formations of absorption peaks representing five different functional groups for MNC10-20. The peaks are summarized in Supplementary Table 1 as well. The peak 2325.91 cm^−1^ represents the C–H asymmetric stretching vibration of aliphatic CH_3_ groups^60^. The peak 1463.34–1443.47 cm^−1^ presents the C-H bending of the CH_2_ and the aliphatic group as the palm oil, but the peak started to decrease as time increased. The peak 813.54–875.35 cm^−1^ presents the fingerprint for the alkane group of aromatic CH.

Meanwhile, the peaks 2101.74 cm^−1^ and 1589.18 cm^−1^ present the C–C bond that forms alkyne and C=C aromatic rings, respectively^61^. The small peak of 1695.15 cm^−1^ shows the C=O bond of free fatty acid from the carbonyl group. It originated from the carbonyl group of CPO and ferrocene during the synthesis. The new formation of peaks ranging from 539.04 to 588.48 cm^−1^ is related to the ferrocene's Fe–O vibration bond. Based on the peaks shown in Supplementary Fig. 4, it can be observed that synthesis time could diminish several peaks and the new formation of chemical bonds in the magnetic nanocarbons.

#### Raman spectroscopy analysis

**Figure 4:**
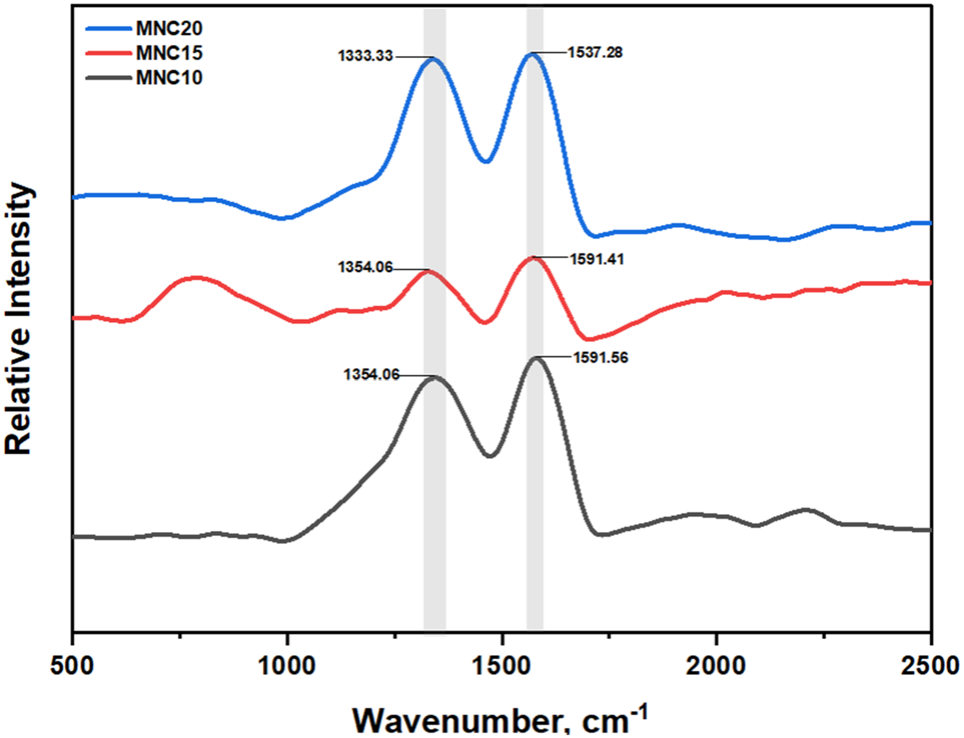
RAMAN spectra of MNC10, MNC15, and MNC20.

Raman spectroscopy analysis using a 514 nm wavelength incident laser of the magnetic nanocarbon prepared with different synthesis times is presented in Fig. 4. All spectra for MNC10, MNC15, and MNC20 consist of two intense bands attributed to the vibrational modes of sp^2^ carbon species with a low sp^3^ carbon content commonly found in defective nano graphitic crystallites^62^. The former peak, around 1333–1354 cm^−1^, represents the D-band, which is unfavoured in the perfect graphite and corresponds to the structural disorder and other impurities^63,64^. The second foremost peak around 1537–1595 cm^−1^ results from the in-plane bond stretching or the crystalline and ordered graphitic shape. However, the peak shifted about 10 cm^−1^ compared to the G-band of graphite, indicating that the MNCs have a low stacking order of sheets and defective structure. The relative intensity of the D-band and G-band (*I*_D_/*I*_G_) ratio is used to estimate the graphite crystallite and the purity of the samples. Based on the Raman spectra analysis, the value of I_D_/I_G_ for all the MNCs ranges around 0.98–0.99, indicating structure defects due to the Sp^3^ hybridization. This situation could explain the presence of the less tense peak 2*θ*: 26.20° for MNC15 and 26.28° for MNC20 in XRD spectra, as shown in Fig. 4, which is assigned to the graphite peak in the JCPDS file. The *I*_D_/*I*_G_ ratio of the obtained MNC in this work is in the range of other magnetic nanocarbon, such as 0.85–1.03 for hydrothermal and 0.78–0.96 for pyrolysis process^65,66^. Hence, this ratio could indicate that the present synthesis method could be used widely.

### Magnetic properties of the magnetic nanocarbons

**Figure 5:**
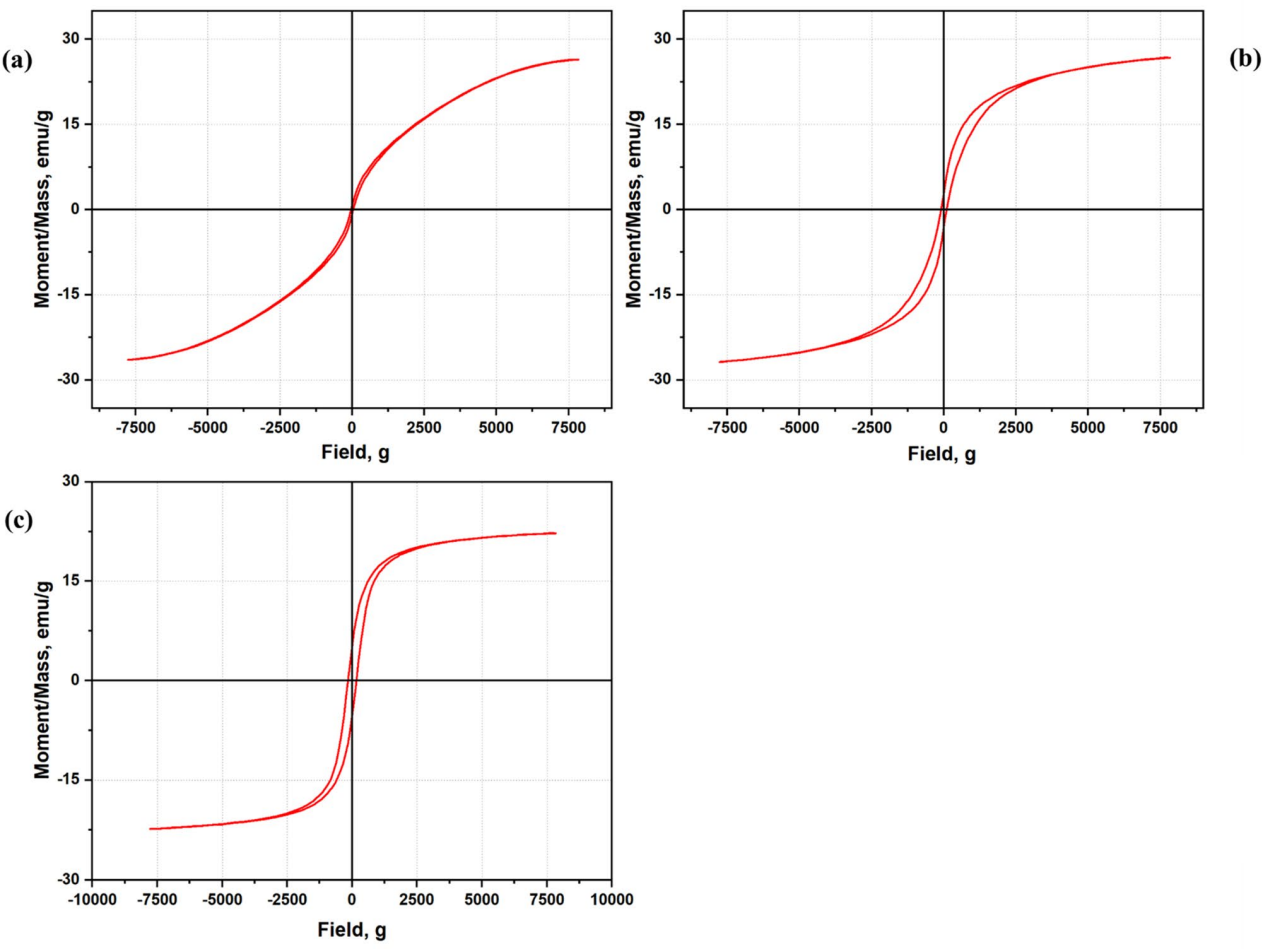
Magnetic hysteresis of (**a**) MNC10, (**b**) MNC15, and (**c**) MNC20.

The magnetic characterization for the MNCs was analyzed using a vibrating sample magnetometer. The obtained magnetic hysteresis is illustrated in Fig. 5. Generally, the MNC acquires magnetic properties from the ferrocene during the synthesis time. These additional magnetic properties can enhance the adsorption capacity of the nanocarbon later. As shown in Fig. 5, the samples can be identified as superparamagnetic materials. According to Wahajuddin & Arora^67^, the superparamagnetic condition is when the samples become magnetized up to their saturating magnetization (MS) when an external magnetic field is applied. Later, the sample no longer exhibits residual magnetic interaction^67^. It is noticed that the saturation magnetization is improved with increasing synthesis time. Interestingly, MNC15 has the highest magnetic saturation due to optimum synthesis time to induce strong magnetic formation (magnetization) when there is an external magnet. This may be due to the presence of Fe_3_O_4_, which demonstrated more excellent magnetic properties than other iron oxides such as a ɣ-Fe_2_O. The MNC’s adsorptive saturation moment per unit mass is in the order of MNC15 > MNC10 > MNC20. The magnetic parameters obtained are tabulated in Table 2.

**Table 2:** VSM parameters for different MNC.

MNC	Magnetization, Ms (emu/g)	Retentivity, Mr (emu/g)	Coercivity, Hci (g)
MNC10	26.476	0.73126	35.116
MNC15	26.835	3.0330	93.885
MNC20	22.32	5.3329	161.33

The minimum magnetic saturation value using a conventional magnet in the magnetic separation procedure was around 16.3 emu g^−1^. The potential of the MNC to remove contaminants such as dyes in the aqueous environment and the easy removal of the MNC becomes adding factor to the produced nanocarbon. The study shows that the magnetic saturation of the MNC was considered high. Thus, the magnetic saturation values achieved by all the samples are more than sufficient for the magnetic separation procedure.

### Proposed mechanism for reaction formation of magnetic nanocarbons

Recently, metal strips or wires have been gaining attention as catalysts or dielectric media during microwave synthesis. The metal-microwave reaction could generate a high temperature or reaction inside the reactor. This study believes the tips and condition (coiled) stainless steel wire promote microwave metal discharge and heating. The stainless steel has sharp irregularities at the tips, which induced the surface charge density and the external electrical field to reach high values. When the charges accumulate enough kinetic energy, the charged particles will jump out of the stainless steel, causing the ionization of the surrounding medium and forming an electric discharge or sparks^68^. The metal discharge significantly assisted in the cracking reaction of the solutions accompanied by high-temperature hot spots. Based on the temperature graph in Supplementary Fig. 2b, the temperature escalates rapidly, marking the existence of high-temperature hotspots other than the intense discharge phenomena.

Simultaneously, the heating effect is observed as loosely bounded electrons can move and concentrate at surfaces and points^69^. As the stainless steel is coiled, the high surface area of the metal inside the solutions promotes the induced eddy current on the material’s surface and supports the heating effects. This condition effectively helps crack the CPO and the ferrocene's long carbon chain and the ferrocene. The constant temperature rate, as seen in Supplementary Fig. 2b, indicates that a uniform heating effect is observed in the solution.

The proposed mechanism of MNC formation is shown in Supplementary Fig. 7. The long carbon chain of CPO and the ferrocene starts to crack at high temperatures. The oil decomposed, forming cracked hydrocarbons that become the precursors of carbon nuclei, referred to as some small spheres inside the FESEM images in MNC10^70^. Due to the energy surrounding and pressure at atmospheric conditions^71^. Meanwhile, the ferrocene also exhibits cracking, forming catalysts for the carbon atoms deposited on the Fe. Then, fast nucleation occurs, and the carbon nuclei oxidize, forming amorphous and graphitic carbon layers on top of the nuclei. As time increases, the sizes of the spheres become more precise and more uniform. Simultaneously, the existing Van der Waals forces also lead to the agglomerated collection of spheres^52^. As the Fe ions are reduced into Fe_3_O_4_ and ɣ-Fe_2_O_3_ as identified in XRD analysis, different types of iron oxide form onto the nanocarbon's surface, forming magnetic nanocarbons. The EDS mapping shows that the Fe atoms are distributed firmly on the surface of the MNC, as seen in Supplementary Fig. 5a–c.

Differently, for 20 min of synthesis time, the carbon becomes aggregated. It develops larger pores on the surface of the MNC, providing that the MNC could be considered activated carbon, as shown in FESEM images in Fig. 1e–g. This difference in pore size could be due to the contribution of the iron oxide from the ferrocene. At the same time, several distorted flakes are present due to the high temperature achieved. The magnetic nanocarbon exhibits various morphologies during different synthesis times. The nanocarbon is more likely to develop a spherical shape at a low synthesis time. Meanwhile, pores and flakes are achievable, although the differences in synthesis time are only 5 min in range.

### Adsorption properties of magnetic nanocarbon

Magnetic nanocarbon has the potential to remove contaminants in an aqueous environment. Their ability to be easily removed after being utilized is an added factor in using the nanocarbon produced in this work as adsorbents. In exploring the adsorption properties of magnetic nanocarbons, we have studied the ability of the MNC to decolorize the Methylene Blue (MB) solution at 30 °C without any pH adjustment. Some studies concluded that the performance of the carbon absorber in the 25–40 °C range does not have any significant role in determining MB removal. Although extreme pH values play an essential role, generating electrical charges on the surface functional groups can occur, leading to interference in adsorbate-adsorbent interaction and affecting adsorption. Thus, the mentioned conditions were selected in this study by considering these situations and the necessity for typical wastewater treatment.

**Figure 6:**
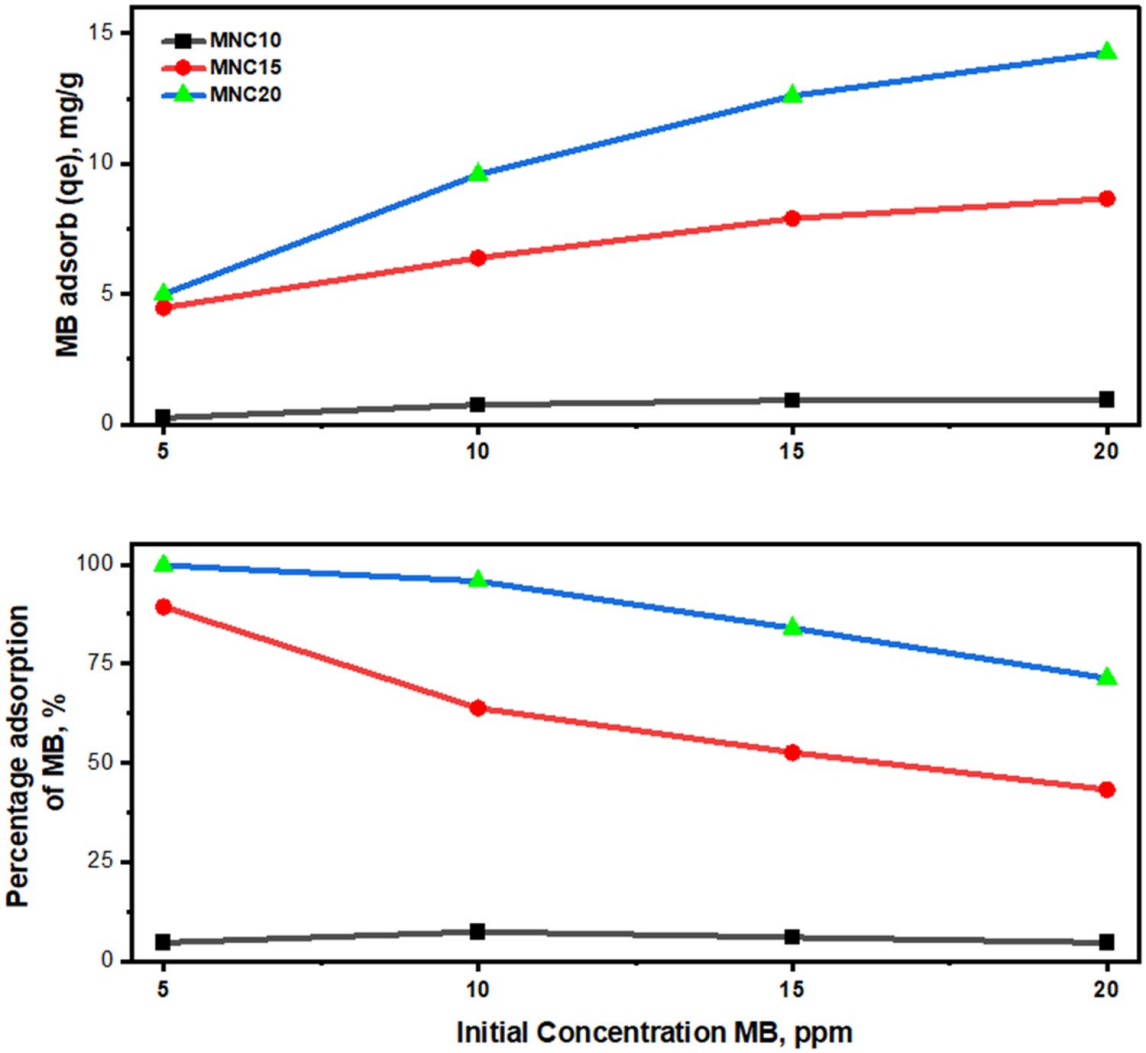
MB adsorption capacity (mg/g) and percentage of MB removal for MNC10-MNC20.

In this work, batches of adsorption experiments were performed by adding 20 mg of MNC to 20 ml of Methylene Blue aqueous solutions with different standard initial concentrations (5–20 ppm) at a fixed contact time^60^. The condition of methylene blue solution for different concentrations (5–20 ppm) before and after treatment with MNC10, MNC15, and MNC20 are shown in Supplementary Fig. 8. The colour level of the MB solution was found to diminish when different MNCs were used. Interestingly, it was observed that MNC20 had easily decolorized the MB solution at a 5 ppm concentration. Simultaneously, MNC20 also decreased the colour level of MB solution compared to other MNCs. The UV–Vis spectra for MNC10-20 are demonstrated in Supplementary Fig. 9. Meanwhile, the removal rate and adsorption information are shown in Fig. 6 and Table 3, respectively.

**Table 3:** Adsorption properties, including Langmuir and Freundlich for the magnetic nanocarbons.

	Adsorption capacity, qe (mg/)g				Percentage removal, (%)				Langmuir			Freundlich		
	5 ppm	10 ppm	15 ppm	20 ppm	5 ppm	10 ppm	15 ppm	20 ppm	*q*_max_ (mg/g)	K_L_	*R*^2^	1/n	K_F_	*R*^2^
MNC10	0.2364	0.7456	0.9136	0.9442	4.73	7.46	6.091	4.72	− 2.4705	− 0.0191	0.9039	1.0199	0.0572	0.8081
MNC15	4.4668	6.3801	7.8942	8.6570	89.34	63.80	52.63	43.29	8.1393	2.2415	0.8867	0.2213	5.0502	0.9797
MNC20	4.9873	9.5788	12.59394	14.2611	99.75	95.79	83.96	71.31	12.04819	55.3333	0.9286	0.1731	10.7730	0.9960

Strong peaks of Methylene blue can be found at 664 nm and 600 nm. Generally, the intensity of the peaks gradually shrank with the decrement of the initial concentration of MB solutions. Supplementary Fig. 9a shows UV–vis spectra of the MB solutions with different concentrations after being treated with MNC10, which only slightly changes the peak’s intensity. On the other hand, MB solutions' absorbance peaks significantly decreased after being treated with MNC15 and MNC20, as shown in Supplementary Fig. 9b and c, respectively. These changes can be seen clearly as the concentration for the MB solution decreased. Nevertheless, the changes in the spectra achieved by all the three magnetic carbons are sufficient and can afford to remove Methylene Blue dyes.

Based on Table 3, the results obtained for the number of MB adsorb and percentage of MB adsorption were plotted in Fig. 6. The MB adsorption increases as a more significant initial concentration are used for all the MNCs. Meanwhile, the percentage of adsorption or MB removal (MBR) shows the opposite trend when the initial concentration increases. Unoccupied active sites exist on the adsorbent surface at a lower initial concentration of MB. Increasing the dye concentration, the unoccupied active sites for adsorption of the dye molecules will decrease. Others concluded that saturated active bio adsorbent sites would be reached during that condition^72^.

Unfortunately, for MNC10, the MBR increases and diminishes after 10 ppm of MB solution. At the same time, only a tiny percentage of MB are adsorbed. This indicated that 10 ppm is the optimum concentration to be adsorbed by MNC10. For all MNCs studied in this current work, the order of the adsorption capacity was as follows: MNC20 > MNC15 > MNC10; with average values of 10.36 mg/g, 6.85 mg/g, and 0.71 mg/g; and average values of MB removal of 87.79%, 62.26%, and 5.75%, respectively. Therefore, by considering adsorptive capacity and the UV–Vis spectra, MNC20 shows the best adsorption properties among the synthesized magnetic nanocarbons. Although the adsorption capacity is lower compared to other magnetic nanocarbon such as magnetic MWCNT composite (11.86 mg/g) and halloysite nanotube-Fe3O4 magnetic nanoparticle (18.44 mg/g), this study does not require additional usage of harsh chemicals as the catalyst, provide clean and feasible synthesis method^73,74^.

As shown by SBET values of the MNCs, the high specific surface area provides more active sites for the MB solution's adsorption. It becomes one of the essential features offered by synthesized nanocarbons. At the same time, as the MNC is smaller in size and the synthesis time is tolerable and short, it complies with the essential qualities of a promising adsorbent^75^. Compared to the conventional natural adsorbent, the synthesized MNC has magnetic saturation that can easily be removed from the solution under an external magnetic field^76^. Hence, shorten the time required for the whole treatment process.

#### Adsorption isotherms of the MNCs

**Figure 7:**
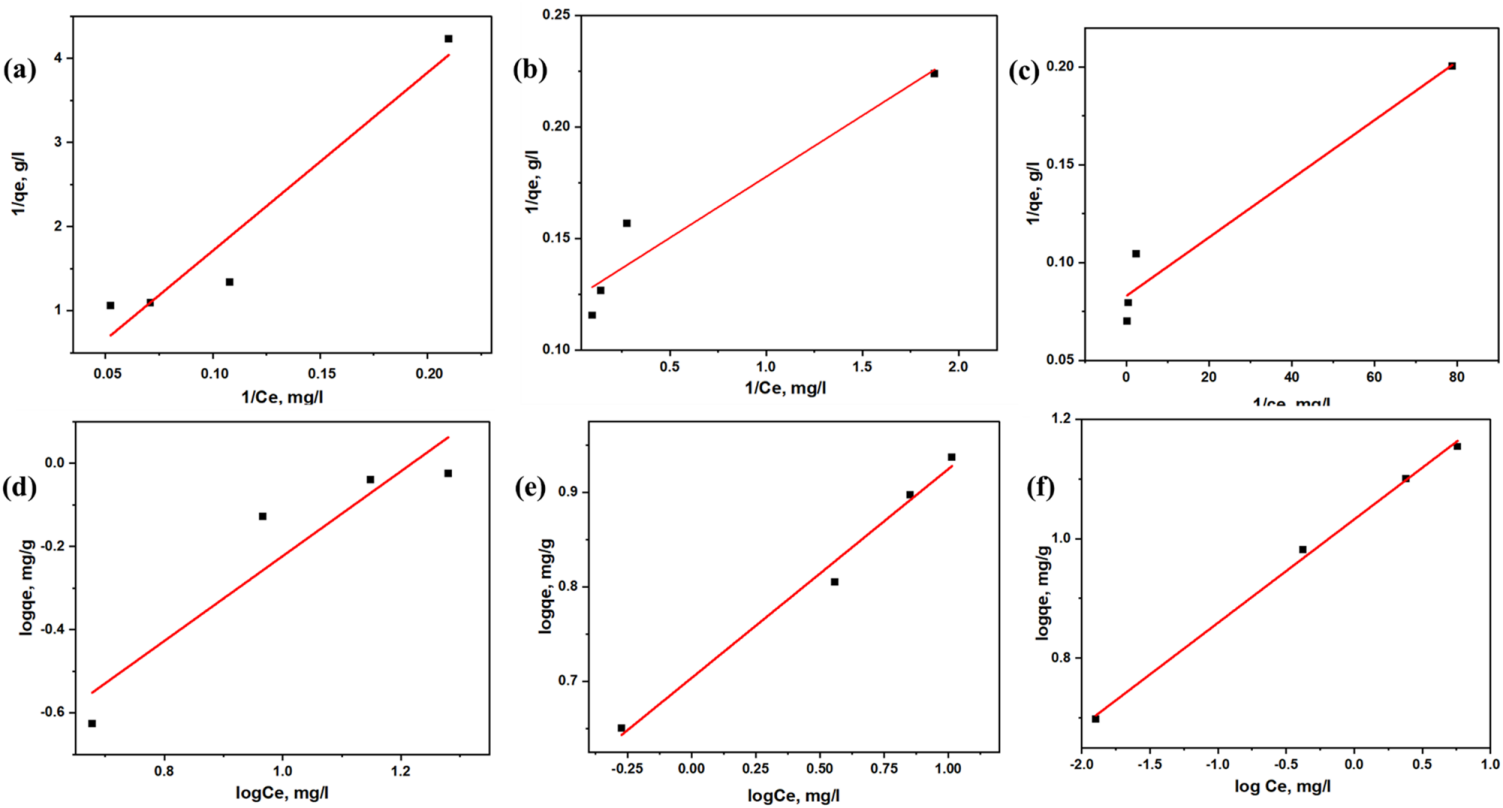
The isotherm models for (**a**–**c**) Langmuir Isotherm and (**d**–**f**) Freundlich Isotherms of MNC10, MNC15 and MNC20.

Adsorption isotherm is necessary for understanding the adsorption process and later demonstrates how the adsorbate is distributed between the liquid–solid phases when it reaches equilibrium. The standard isotherm equations used are Langmuir and Freundlich equations, which explain the adsorption mechanisms, as presented in Fig. 7. The Langmuir model can well show the formation of monolayer adsorbate on the outer surface of the adsorbent. The isotherm is best described as a homogenous adsorption surface. Meanwhile, Freundlich isotherms best conclude that several sites of the adsorbent and adsorption energies are involved in pressing adsorbate over the heterogenous surfaces.

Adsorption isotherms at low solute concentrations are often linear^77^. The linear expression of the Langmuir isotherm model can be represented by Eq. 1 to determine the adsorption parameter.

1 $$\frac{1}{qe} = \frac{1}{{{\text{KL}}q\max }} + \frac{1}{Ce} + \frac{1}{q\max }$$

K_L_ (L/mg) represents Langmuir’s constant, representing the MB and MNC binding affinity. Meanwhile, qmax represents the maximum adsorption capacity (mg/g), qe represents the concentration of the adsorbed MB in mg/g, and Ce represents a concentration of the equilibrium of the MB solution. The linear expression of the Freundlich isotherm model can be described as follow:

2 $$\log qe = Log{\text{K}}f + \frac{1}{n}LogCe$$

K*f* is the Freundlich constant used to measure the adsorption capacity, and 1/n represents the adsorption intensity.

The calculated values from Langmuir and Freundlich isotherm parameters with the correlation coefficient (*R*^2^) are presented in Table 3. The analyzed results demonstrated that the *R*^2^ values from all the Freundlich models were superior (*R*^2^ > 0.90) to the Langmuir model, indicating that the adsorption behaviour was better discussed and described by the Freundlich isotherm. Hence, it verified that the existing system is heterogeneous. These situations are related to the presents of different chemical and structural properties on the surfaces of carbon spheres. The heterogeneity factor, or nF, can be used to indicate whether the adsorption is favourable to linear (nF = 1), chemical process (nF < 1), or whether the physical process (nF < 1)^78^. Meanwhile, 1/nF < 1 indicates normal adsorption, and 1/nF > 1 indicates cooperative adsorption^79^. Generally, the MNC shows nF > 1 and 1/nF, which summarized that the adsorption of MB is favourable for the physical process and normal adsorption^78,80^.

The Langmuir constant K_L_ corresponds to the energy involved in the adsorption process. It is known that a high K_L_ value is associated with a high adsorption rate between the surface of adsorbents and dye molecules. The estimated K_L_ values for the MNC are in the order of MNC20 > MNC15 > MNC10, indicating the interactions between MNC20 and Methylene Blue were the highest, while MNC10 and Methylene Blue were the lowest. But due to the negative values from the Langmuir parameter, q_max_ and low value of RL shows that the Langmuir isotherm model does not favorably describe the adsorption of the MNC produced. All isotherm parameters for both isotherms are presented in Table 3, having the R^2^ values of the Freundlich isotherm greater than those from the Langmuir model for all MNCs. Thus, it defines that the Freundlich isotherm is suitable for describing the adsorption equilibrium of MB onto MNC.

## Conclusion

Crude palm oil was successfully converted into magnetitic nanocarbon via a microwave-assisted arc in the liquid method. Metal arcing under microwave irradiation was able to cause cracking of the long hydrocarbon chain in palm oil. At the same time, ferrocene was interpreted to form nuclei and act as catalysts for the carbon atoms deposited on top of it. Additionally, ferrocene is reduced into Fe_3_O_4_ and ɣ-Fe_2_O_3_ as identified in XRD analysis, and different types of iron oxide form on the surface of the deposited carbons. The synthesized MNCS exhibited good adsorbent properties in removing MB for wastewater treatment. High BET surface area and good magnetic saturation of MNC20 showed the best adsorption capacity based on the calculated maximum adsorption capacity, followed by MNC15 and MN10. The R^2^ calculated in the Freundlich theorem also supports the magnetic nanocarbon's ability to adsorb the cationic dyes. This method provides several advantages, including (i) simple, convenient, and feasible to operate, (ii) the cracking reaction has instantly occurred as the microwave irradiation is started, and (iii) does not need to use harsh chemicals to purify the samples.

## Supplementary Information

Supplementary Information.

## Data Availability

The datasets used and analysed during the current study are available from the corresponding author on reasonable request.
