# Supplementary material for: Synthesis of magnetic nanocarbon using palm oil as the green precursor via microwave-assisted arc for wastewater treatment
Source: Sci Rep. 2022 Nov 4;12:18698. doi: 10.1038/s41598-022-21982-y (PMC9636159; doi:10.1038/s41598-022-21982-y)
Supplement: Supplementary file 1 — Supplementary Information. [file 41598_2022_21982_MOESM1_ESM.docx]

**Synthesis of Magnetic Nanocarbon Using Palm Oil as the Green Precursor Via Microwave-Assisted Arc for Wastewater Treatment**

Nurul Zariah Jakaria Zakaria ^1*^, Shaifulazuar Rozali ^1*^, Nabisab Mujawar Mubarak^2*^, Mohammad Khalid Siddiqui ^3^

^1^Department of Mechanical Engineering, Faculty of Engineering, University of Malaya, 50603, Kuala Lumpur, MALAYSIA,

^2^ Petroleum and Chemical Engineering, Faculty of Engineering, Universiti Teknologi Brunei, Bandar Seri Begawan BE1410, Brunei Darussalam,

^3^ Graphene & Advanced 2D Materials Research Group (GAMRG), School of Engineering and Technology, Sunway University, No. 5, Jalan University, Bandar Sunway, 47500, Subang Jaya, Selangor, Malaysia

*Corresponding author email id: [nzariah@um.edu.my](mailto:nzariah@um.edu.my); [azuar@um.edu.my](mailto:azuar@um.edu.my); mubarak.yaseen@gmail.com

**Supplementary Fig. 1**


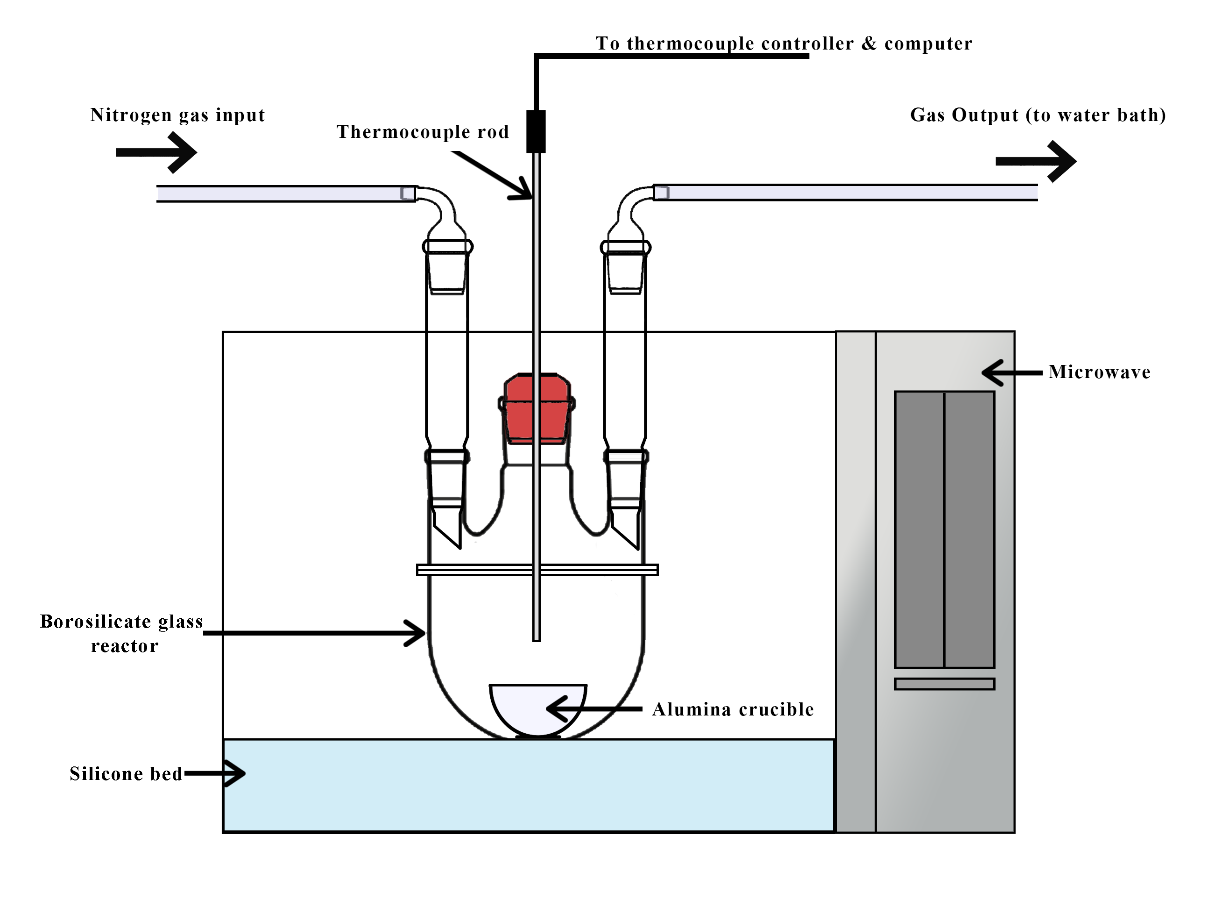


Fig. 1 Schematic drawing of the reactor for a microwave-assisted arc in the liquid

**Supplementary Fig. 2**

**
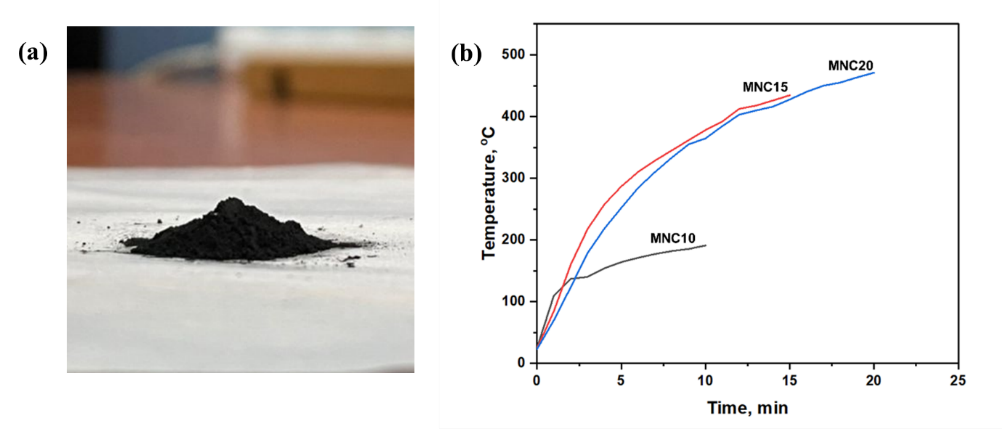
**

**Fig.2 (a) MNC sample and (b) Graph of synthesis temperature vs. time for the produced magnetic nanocarbons**

**Supplementary Fig. 3**
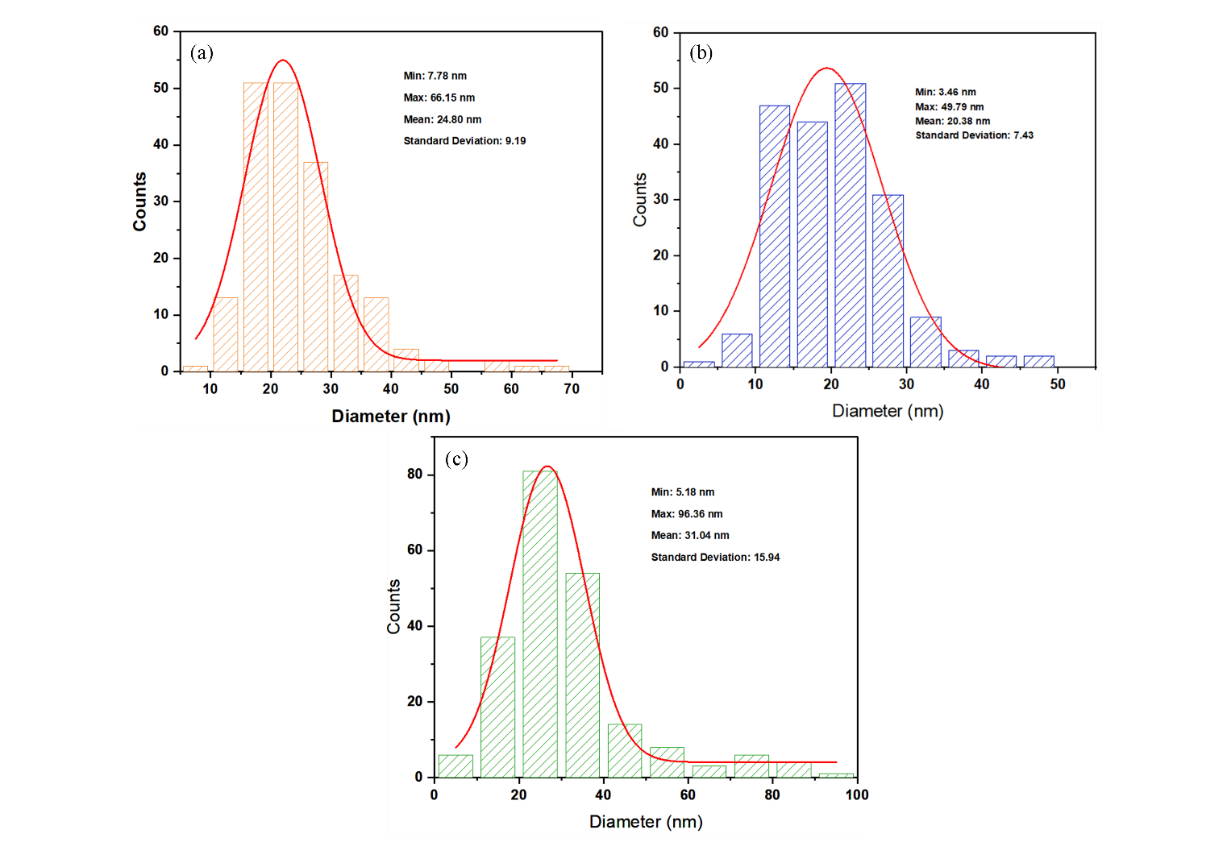


**Supplementary Fig.3 Histogram size distribution of (a) MNC10, (b) MNC15, and (c) MNC20**

**Supplementary Fig.4**


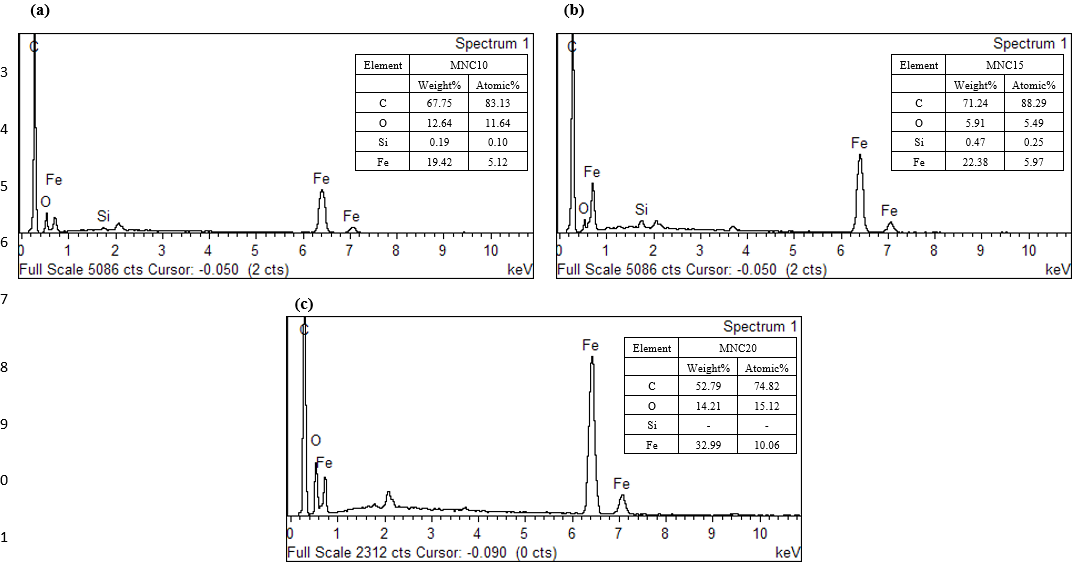


**Supplementary Fig. 4 EDS spectra of (a)MNC10, (b)MNC 15, and (c)MNC20**

**Supplementary Fig. 5(a-c)**


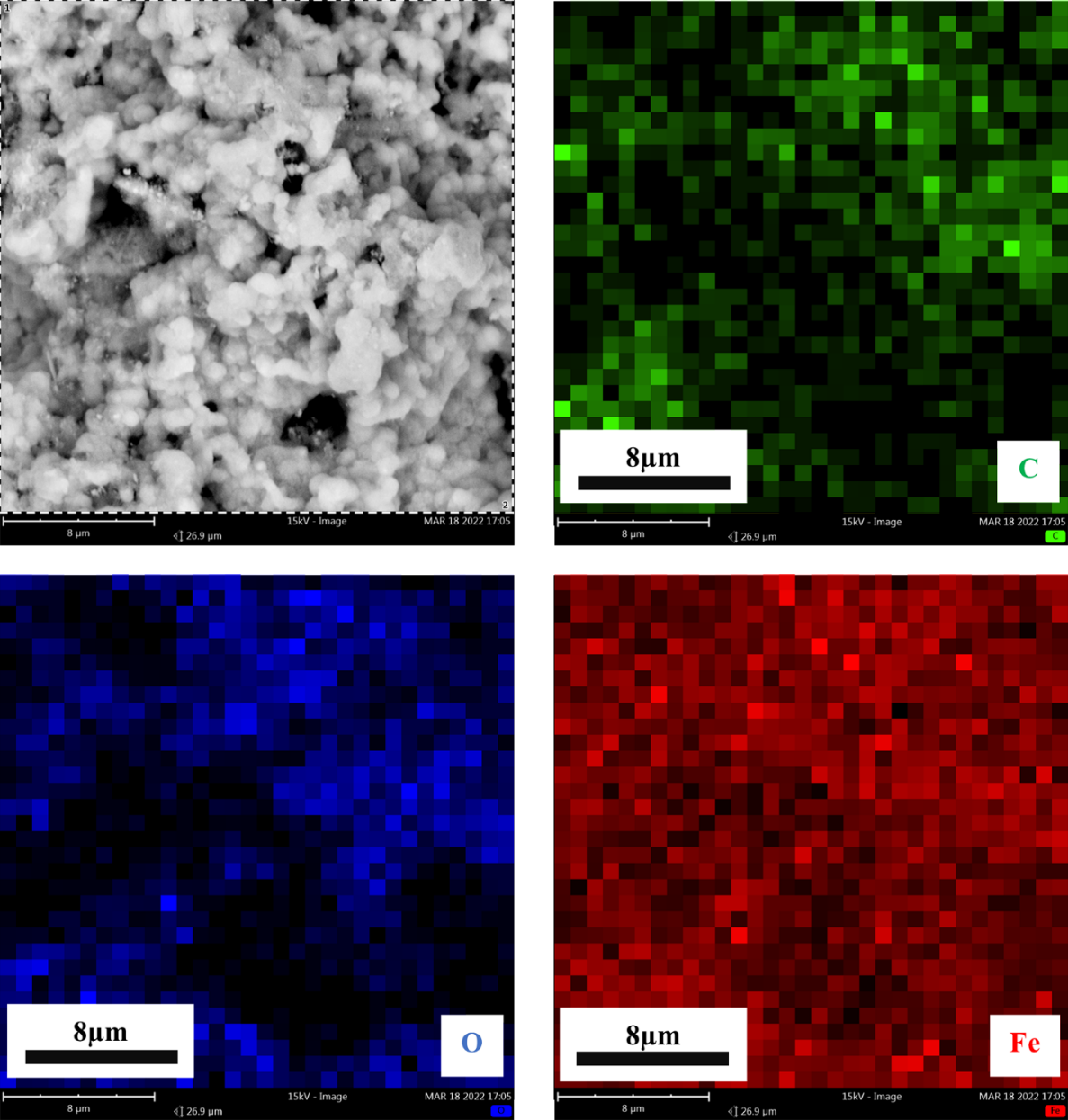


**Supplementary Fig. 5 (a) SEM-EDS mapping of surfaces for MNC10**


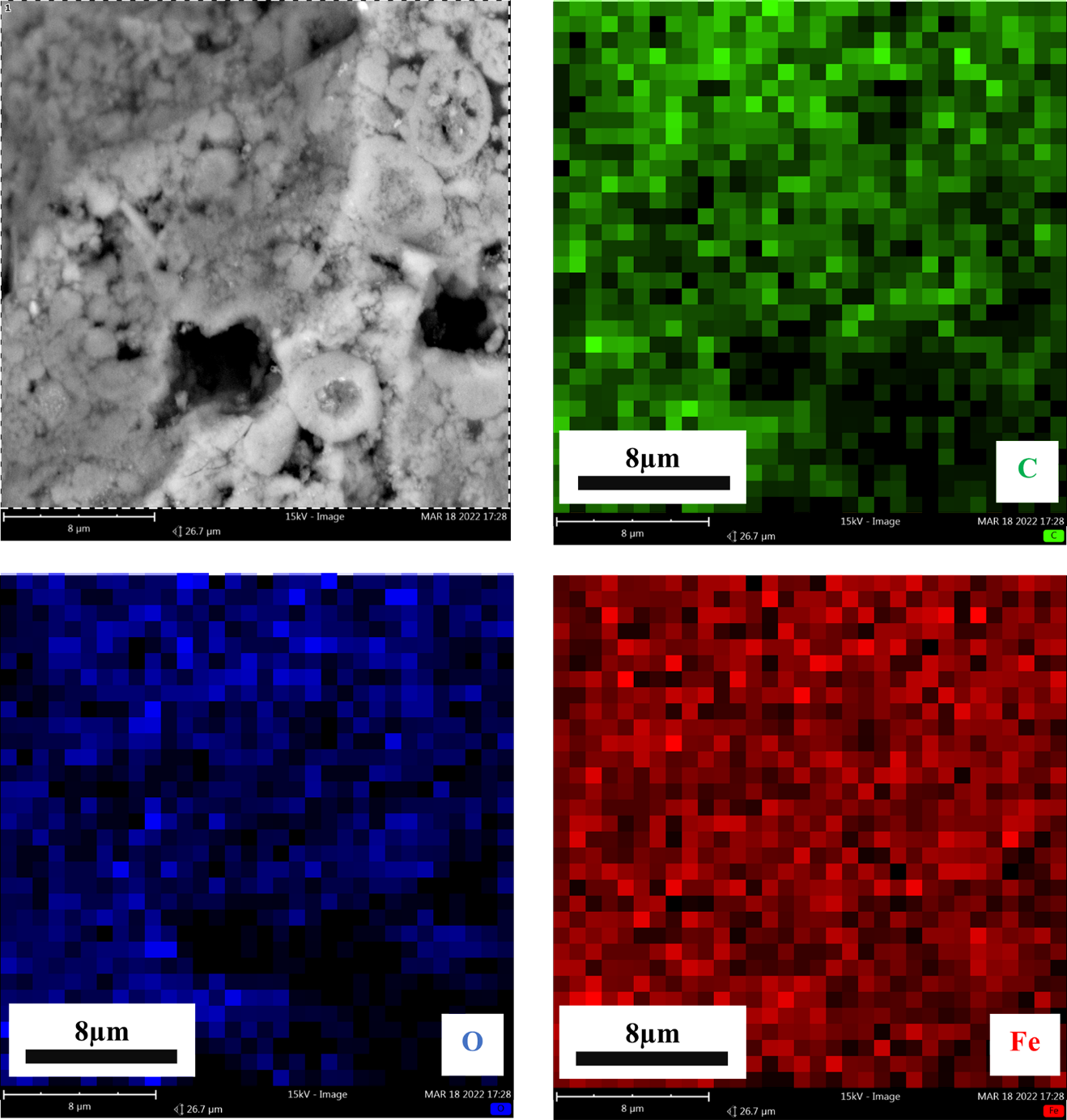


**Supplementary Fig. 5 (b) SEM-EDS mapping of surfaces for MNC15**


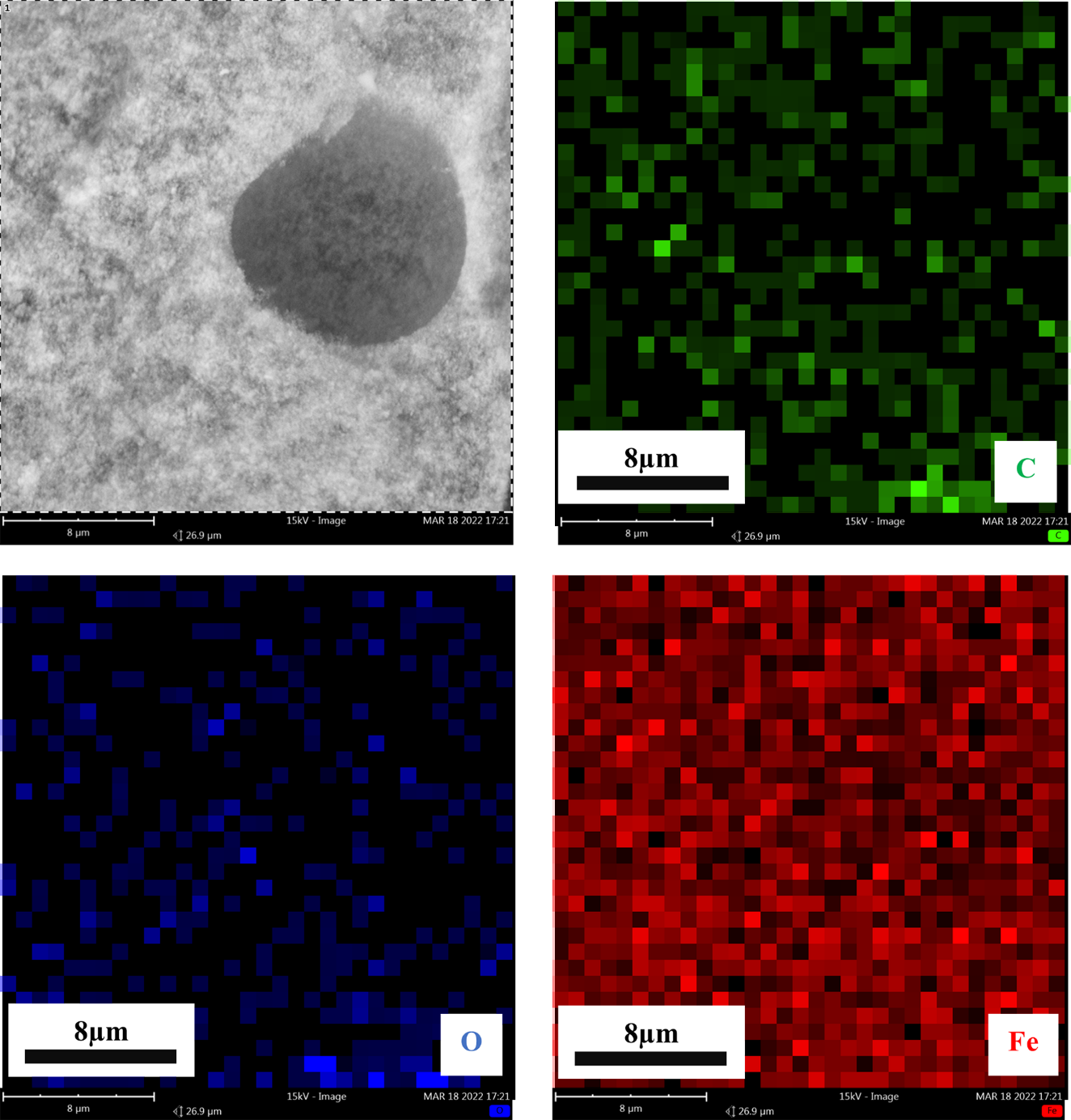


**Supplementary Fig. 5 (c) SEM-EDS mapping of surfaces for MNC20**

**Supplementary Fig. 6**
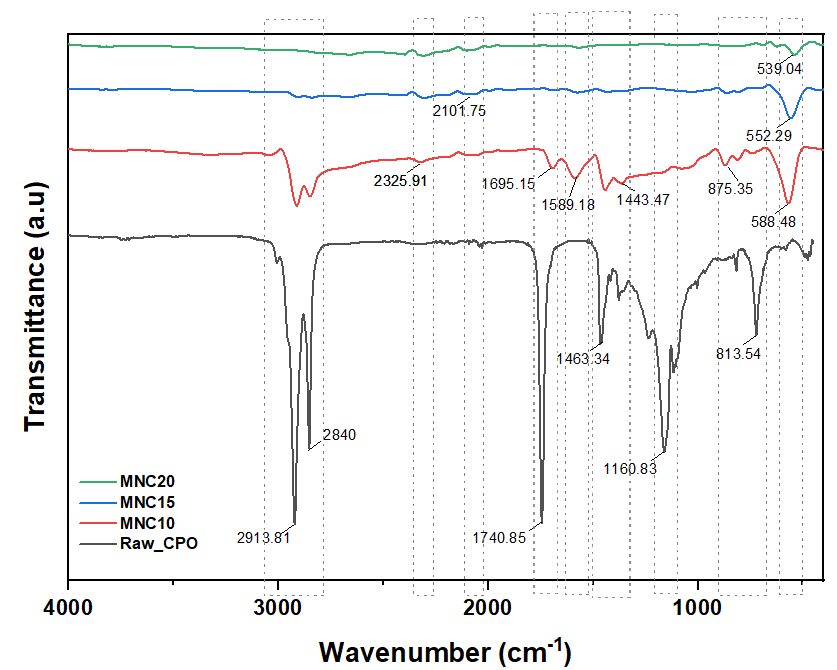


**Supplementary Fig. 6 FTIR spectra of MNCs and raw crude palm oil and MNC10, MNC15 and MNC20**

**Supplementary Fig. 7**


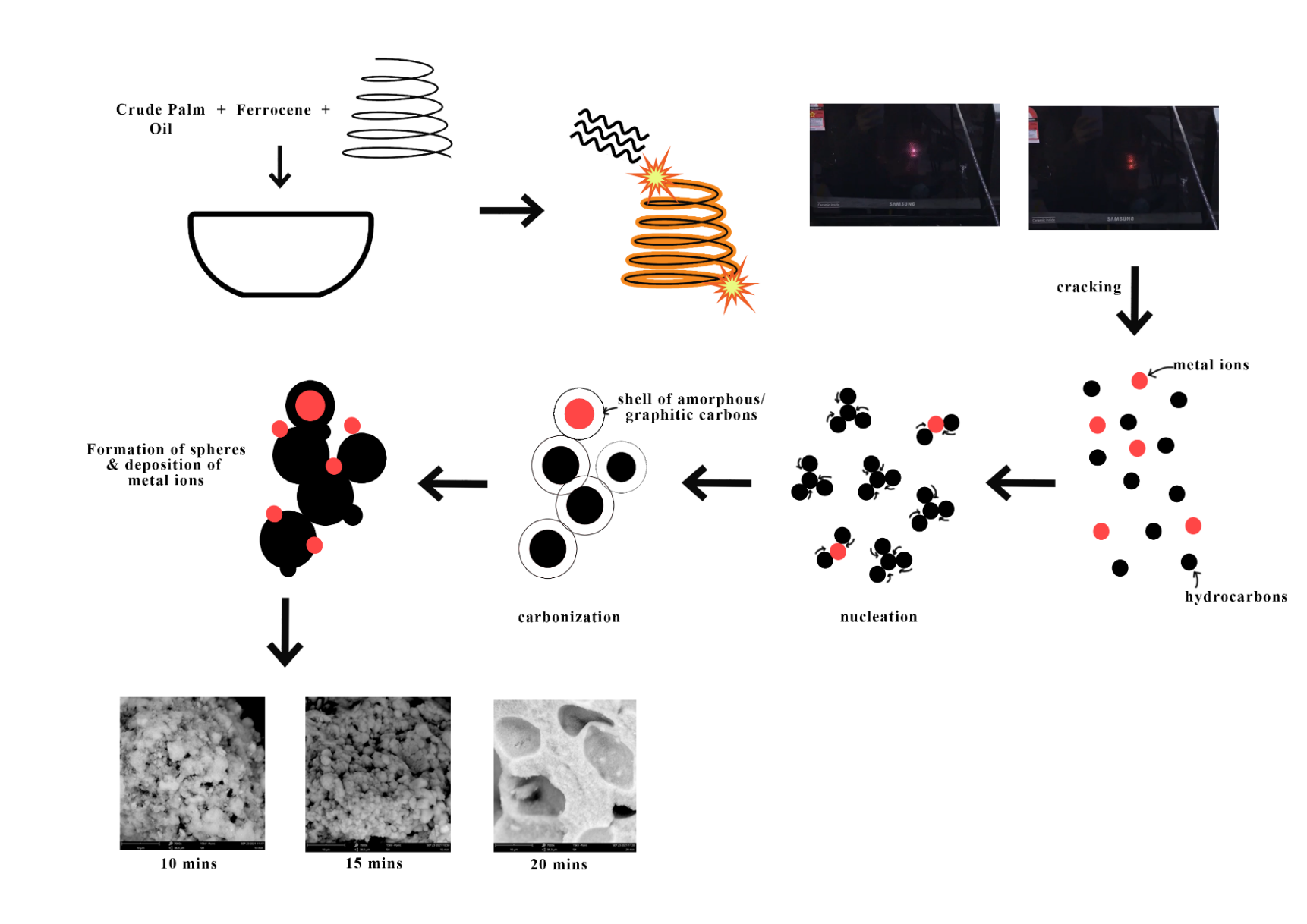


**Supplementary Fig. 7 Proposed mechanisms of magnetic nanocarbon formation by microwave arcing synthesis**

**Supplementary Fig. 8**


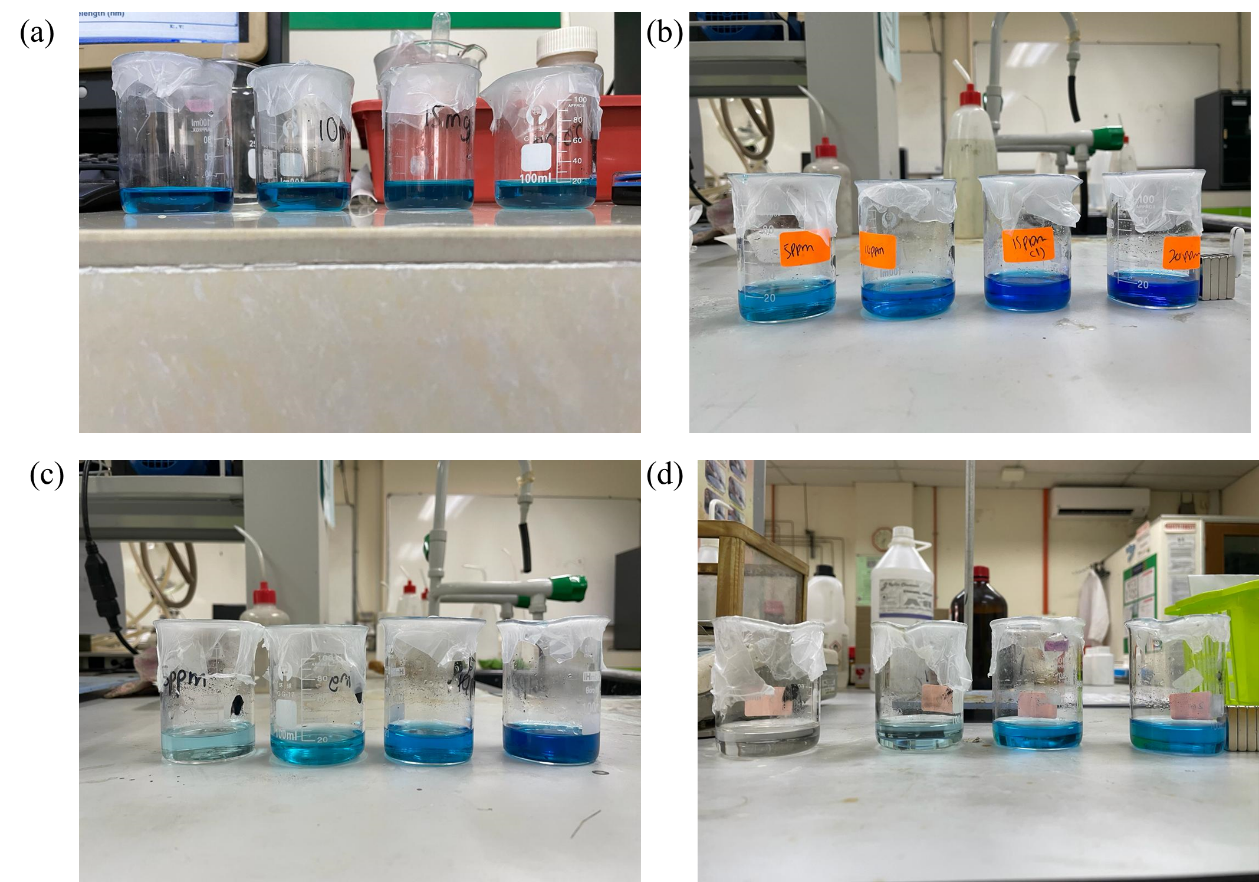


**Supplementary Fig. 8 Methylene Blue solution for (a) before (from left: 5ppm, 10ppm, 15ppm and 20 ppm) and after treated with (b) MNC10, (c) MNC15, and (d)MNC20 (from left: 5ppm, 10ppm, 15ppm and 20 ppm**

**Supplementary Fig. 9**
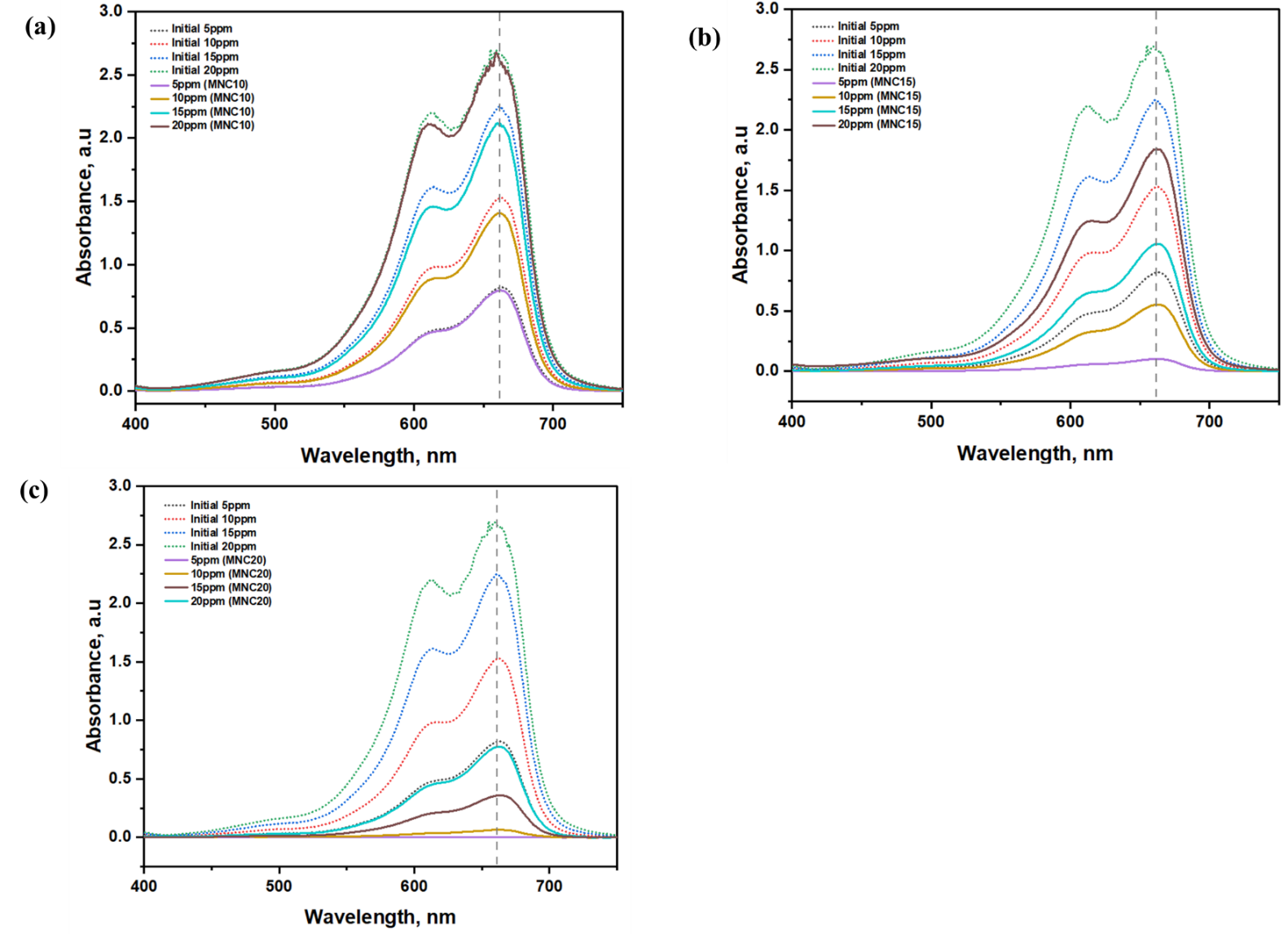


**Supplementary Fig. 9 UV-Vis spectra for MB adsorption after being treated with (a) MNC10, (b) MNC15, and (c)MNC20.**

**Supplementary Table 1**

**Supplementary Table 1 Summary of the chemical bonding in CPO and MNC FTIR spectra**

| FTIR Peaks, cm ^-1^ | | | | Functional Group |
| --- | --- | --- | --- | --- |
| CPO | **MNC10** | **MNC15** | **MNC20** |  |
| 2913.81 | 2913.81 | - | - | CH-alkane/aliphatic group |
| 2840 | 2840 | - | - | CH-alkane |
| - | 2325.91 | 2325.91 | 2325.91 | C-H of aliphatic (CH3) |
| - | 2101.75 | 2101.75 | 2101.75 | C-C alkyne |
| 1740.85 | - | - | - | C=O esters of triglycerides |
| - | 1695.15 | 1695.15 | 1695.15 | C=O carbonyl group |
| - | 1589.18 | 1589.18 | 1589.18 | C=C aromatic ring |
| 1463.34 | 1443.47 | 1443.47 | 1443.47 | C-H bending of CH2 & CH3 aliphatic group |
| 1160.83 | - | - | - | C-O esters |
| 813.54 | 875.35 | 875.35 | 875.35 | CH-alkane |
| - | 588.48 | 552.29 | 539.04 | Fe-O bond |
